# Twitter as a Potential Disaster Risk Reduction Tool. Part II: Descriptive Analysis of Identified Twitter Activity during the 2013 Hattiesburg F4 Tornado

**DOI:** 10.1371/currents.dis.f2e5b9e979af6174d2f97c1f0349be5c

**Published:** 2015-06-29

**Authors:** Guy Paul Cooper, Violet Yeager, Frederick M. Burkle, Italo Subbarao

**Affiliations:** College of Osteopathic Medicine, William Carey University, Hattiesburg, Mississippi, USA; College of Osteopathic Medicine, William Carey University, Hattiesburg, Mississippi, USA; Harvard Humanitarian Initiative, Harvard University, Cambridge, Massachusetts; The Woodrow Wilson International Center for Scholars, Washington, DC, USA; College of Osteopathic Medicine, William Carey University, Hattiesburg, Mississippi, USA

**Keywords:** Communications, Disaster analysis, Disaster risk reduction, Prevention and preparedness, Social media, Twitter

## Abstract

Background: This article describes a novel triangulation methodological approach for identifying twitter activity of regional active twitter users during the 2013 Hattiesburg EF-4 Tornado.

Methodology: A data extraction and geographically centered filtration approach was utilized to generate Twitter data for 48 hrs pre- and post-Tornado. The data was further validated using six sigma approach utilizing GPS data. Results: The regional analysis revealed a total of 81,441 tweets, 10,646 Twitter users, 27,309 retweets and 2637 tweets with GPS coordinates.

Conclusions: Twitter tweet activity increased 5 fold during the response to the Hattiesburg Tornado.  Retweeting activity increased 2.2 fold. Tweets with a hashtag increased 1.4 fold. Twitter was an effective disaster risk reduction tool for the Hattiesburg EF-4 Tornado 2013.

## Background

This is Part II of the four part series of articles that analyze the effectiveness of Twitter as a disaster risk reduction tool in mitigating morbidity and mortality. Part I reviewed the relevant background for Twitter, its terminology, existing methodological challenges, and provided a novel methodological approach that shows greater validity and reliability.^1^ This part describes the detailed methodological application of the novel triangulation methodology used to filter the ‘haystack ‘of tweets transmitted during the 2013 Hattiesburg Tornado among those captured from the over 2 billion tweets in the 96 hour window of the storm that were emitted on the Twitterverse.^2^ The data generated from the approach provides a descriptive analysis of the regional Twitter activity 48 hours pre- and post- Hattiesburg Tornado. Part III describes the ‘needle in the haystack’ itself by identifying the Top 100 Twitter Users that were re-tweeted 48 hours pre- and post -tornado and analyzing the significant statistical relationship between metadata variables and in particular disaster-related hashtags to the users themselves.

## Methodology

Part I described the novel methodological approach. This first step includes data extraction of all tweets utilizing PowerTrack rules from GNIP (an authorized reseller of Twitter data) that include broad-based tornado disaster centric filters.^3^ The second step uses a triangulated approach to filter and identifies regional Twitter users. The methodology employed a filtration approach to ‘capture or triangulate’ tweets from multiple angles (location, biography, retweets of Twitter users) to ensure they were actually utilized by the at-risk disaster affected geographic population (Figure 1). This structured analysis utilized six sigma principles and was validated by an independent quality analysis team.

**Filtration Methodology:**
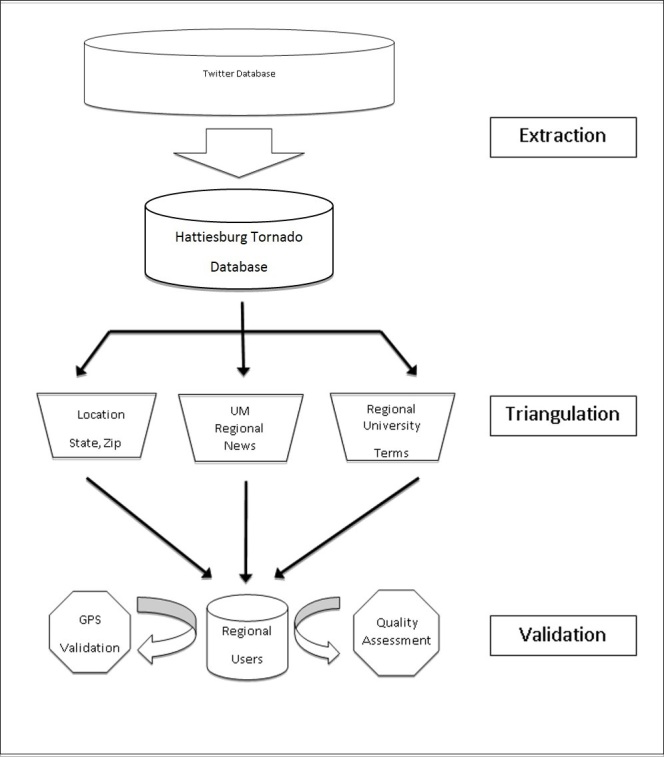
Tweets were extracted and user region was determined through triangulation followed by GPS and quality assessment validation.

**TWITTER DATA EXTRACTION**

Tweets were extracted from the Twitter database through an authorized Twitter data reseller, GNIP, using filters, and PowerTrack rules that were defined by an 11 day window, February 5, 2013 5:00 pm to February 15, 2013 5:00 pm based at the date and time of the Hattiesburg Tornado February 10, 2013 5:00 pm (Figure 1).^4^ The PowerTrack rules were based on a set of criteria that included state and local biographical locations, disaster and relief hashtags and keywords, local news media sources, and statewide GPS coordinates.^5^^,^ 6

**TWITTER DATA TRIANGULATION**

The data extraction was completed requiring a tweet metadata or attached profile of one of a variety of previously used disaster hashtags, keywords, Mississippi cities, Mississippi, or geolocation within the state of Mississippi. Data was returned from GNIP as a JSON format and subsequently turned into a tab delimited file, and secondary filtration was done by a Unix-based Perl script on a Lenovo V570 laptop.^7^ Regionality was then established based on a previously verified methodology focusing on Mississippi and Alabama regional users. The data was then divided into two 48 hour windows before and after the tornado impact with a 2 hour pre-tornado buffer to capture tweets just prior to impact. Tweets and users were analyzed based on the criteria found in Figure 1.

**Table 1. Regional User Data:** Regional user tweet data was analyzed 48 hours before and 48 hours after the storm with a 2 hour buffer to capture tweets just prior to impact

		Pre-Storm	Post-Storm
Total:			
	Users	3,145	7,501
	Tweets	27,927	53,514
	Retweets	6,551	20,758
	Tweets with a hashtag	5,763	13,598
	GPS tweets	758	1,879
	Application types	141	192
	Verified users	3	3
	Languages	6	8
Average:			
	User account length	785	846
	Followers	745	779
	Klout	33	34
	Friends	506	504

**DATA TRIANGULATION**

Case insensitive regional filters were developed around the geographical locations, news sources (radio, news, television), and colleges/universities in the area. The filters were then applied to the extracted data based on the users: tweets, biographies, and locations (Figure 1). Regionality was defined as Twitter users who had one of the following criteria in the 11 day span: 1) User mentioned a regional news source in their tweet, 2) entered (Mississippi, Alabama, Hattiesburg, Birmingham, USM, Ole Miss, SMTTT) in their biography, or 3) entered (Mississippi, Alabama, Hattiesburg, Birmingham, MS, AL, a regional Zip code, USM, Ole Miss, SMTTT) in their location (Table 2). The categories were then evaluated to determine the contribution of each of the filters. Time zones were collected from all regionally defined users to determine their potential contribution and activity. The activity of the regionally defined users was then centered on the first touchdown of the Hattiesburg Tornado 48 hours pre-storm and 48 hours post-storm were determined.

**Table 2. Regional User Criteria:** User profile categories and the terms that were used to determine the region of a user. Inclusive criteria was captured by more than one term, while exclusive criteria was captured by a single term.

Category	Terms	Inclusive	Exclusive
Tweet:			
	User Mentioned Regional News	1,938	1,444
Biography:			
	Mississippi, Alabama, Hattiesburg, Birmingham	845	186
	USM, Ole Miss	417	126
	SMTTT	327	74
Location:			
	Mississippi, Alabama, Hattiesburg, Birmingham	4,077	1,826
	MS, AL	4,175	1,882
	ZIP (for all MS & AL)	42	44
	USM, Ole Miss	22	18
	SMTTT	1	0

**REGIONAL USER VALIDATION METHODOLOGY**

Regional users were confirmed and validated in a two-tier approach based upon the available GPS coordinates and an independent quality assessment. GPS coordinates for users that had activated their geo-locations were compared against regionally defined users to confirm their presence in Alabama or Mississippi (Figure 1). Reverse geolocation lookup was done through a Ruby on Rails script in conjunction with a geocoder that accessed the Bing API.^8^^,^ 9 GPS Sample standards were set as a 99% confidence interval and a 3.0% margin of error.

**QUALITY ASSESSMENT METHODOLOGY**

A research team comprised of an epidemiologist and a masters in biomedical science, independent of the coding protocol, evaluated the data to determine if it regionally met the criteria established in Table 2 and if non-regionally was appropriately excluded. The results found no apparent errors or aberrations of those terms. Regional GPS threshold, regional quality assessment, and non-regional quality assessment was set at a 99% confidence interval and 3.0% margin of error.

The study received an IRB exemption for human subjects research from the William Carey University IRB Committee.

## Results

**EXTRACTION & FILTRATION**

The 11 day span of approximately 5.5 billion total tweets were reduced to 1.1 million tweets per PowerTrack filters.^3^ These 1.1 million tweets were further extracted from the Twitter database. Initial evaluation of the results revealed that approximately 800,000 of these tweets were not from an area of tornado impact Petal, Mississippi, but Petaling, Malaysia. These tweets were removed leaving 350,583 tweets and 41,458 users in the 11 day span.

**OVERALL TRIANGULATION RESULTS**

Data was first evaluated around the 96 hour window of the tornado (Table 3). The window showed 127,954 posted tweets, 26,938 total users, but only 81,441 were regionally defined tweets posted by 8,423 regional users with 515 users having activated their GPS setting in Twitter (Table 3).

**Table 3. Tornado Impact Data:** Data from a 96 hour window around the tornado impact.

Category	Total
Total tweets	127,954
Total users	26,938
Regional tweets	81,441
Regional users	8,423
GPS users	515
Regional users with GPS	463
Regional tweets with GPS	2,353
Regional confirmed with GPS	2,290

Preliminary evaluation of Twitter users was obtained via filtration sorting through three categories: tweets, biography, and location of users. The relationship between the filters and the collected Twitter users revealed that the terms were mutually exclusive (a single filter detected the user) or mutually inclusive (more than one filter detected the same user) (Table 2).

The first filter category labeled ‘Tweet’ utilized users that specifically referenced the Twitter username of a regional news media outlet (1,938 inclusive users and 1,444 exclusive users (Table 2). The Tweet filter second category labelled ‘Biography’ used the terms Mississippi, Alabama, Hattiesburg, Birmingham (845 inclusive and 186 exclusive users), USM and Ole Miss (417 inclusive users and 126 exclusive users), and SMTTT (327 inclusive users and 74 exclusive users). The third filter category labeled ‘Location’ used the terms Mississippi, Alabama, Hattiesburg, Birmingham (4,077 inclusive and 1,826 exclusive users), MS, AL (4,175 inclusive and 1,882 exclusive users), all ZIP codes for MS & AL (42 inclusive and 44 exclusive users), USM and Ole Miss (22 inclusive users and 18 exclusive users), and SMTTT (1 inclusive user and 0 exclusive users). The further evaluation also revealed that 1,201 users identified as regional did not enter a biography, and 316 did not enter a location.

**TIME ZONE ANALYSIS**

Time zones from the 8,423 users found to encompass 40 time zones, and 2,268 users did not enter a time zone (Table 4). Central time was listed on 4,268 users profiles, Mountain Time on 680 users, Eastern Time on 618 users, Pacific Time on 200 users, and the other 36 time zones represented 389 users. The variable results of time zones excluded them as being considered as usable regional criteria.

**Table 4. Time Zones of the Regional Users:** 

Time Zones	Users
Central Time (US & Canada)	4,268
Null	2,268
Mountain Time (US & Canada)	680
Eastern Time (US & Canada)	618
Pacific Time (US & Canada)	200
Other	389

**AGGREGATE DATA ANALYSIS**

This was completed in the required 96 hours (4 days) Twitter analysis: pre-storm total users (3,145), total tweets (27,927), total re-tweet (6,551), total tweets with hashtag (5,763), total GPS tweets (758), total Twitter application device types (141), total Verified people (3), total languages (6), average use account length (785 days), average followers (745), average Klout (33), average friends (506).

Post-storm results found total users (7,501), total tweets (53,514), total retweets (20,758), total tweets with hashtag (13,598), total GPS tweets (1,879), total application types (192), total verified people (3), total languages (8), average user account length (846), average followers (779), average Klout (34), and average friends (504)(Table 1) (Figures 2-4).

**Regional Users: Tweets per Hour:**
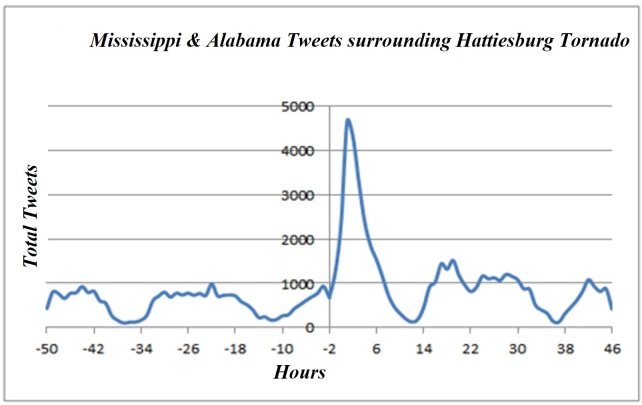
"0" signifies the storm impact. "-50" is 50 hours pre-storm. "46" is 46 hours post-storm. This figure displays the usage of tweets by regionally identified Mississippi & Alabama Twitter users.

**Regional Users: Hashtags per Hour:**
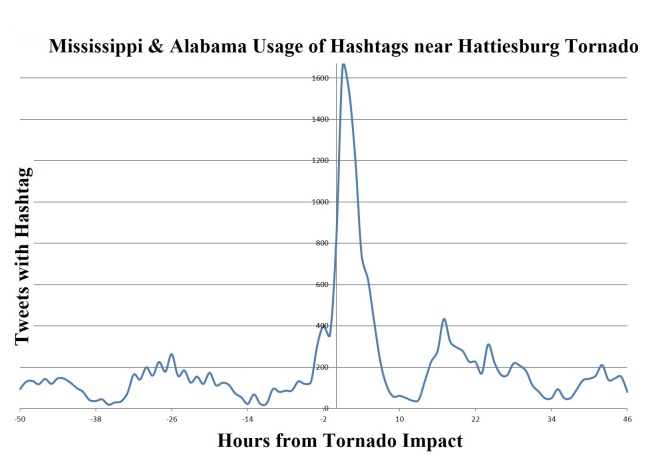
"0" signifies the storm impact. "-50" is 50 hours pre-storm. "46" is 46 hours post-storm. This figure displays the usage of hashtags by regionally identified Mississippi & Alabama Twitter users.

**Regional Users: Retweets per Hour:**
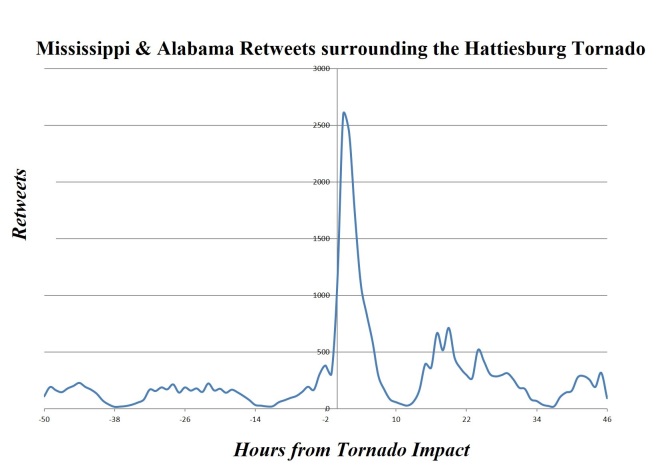
"0" signifies the storm impact. "-50" is 50 hours pre-storm. "46" is 46 hours post-storm. This figure displays the usage of Retweets by regionally identified Mississippi & Alabama Twitter users.

**REGIONAL GPS VALIDATION**

GPS data was provided by 515 total regional users, and 463 users were found to have tweeted with GPS locations in Alabama or Mississippi in the 11 day span. Of the 52 users that did not have GPS locations, 30 users listed Hattiesburg, MS as their location, and 9 listed locations within Mississippi and Alabama. Including those users who may have been traveling and identify themselves as Mississippi or Alabama locations put the likely regional accuracy of 502 of 515, 97.4%. Inspecting the GPS location of the specific tweets 2,290 of 2,353 fall within the state borders. Closer inspection found 41 of the 63 users who tweeted also had their location listed as Hattiesburg, MS.

**QUALITY ASSESSMENT**

Quality assessment of the 900 users showed a 100% validation of no aberrant term usage. Assessing 900 non-regional users found two users that were likely Mississippi or Alabama regional users during the four day window.

## Discussion

The study team successfully developed and validated a novel methodological approach for extracting regional Twitter data despite the anonymity established in popular social media devices. This triangulation methodological approach is designed around the Twitter API or “firehose” in order to provide a real-time or cross-sectional technique to accurately predict user location. Figure 5 provides a categorical summation of our triangulation approach revealing the exclusive terms that provided unique users that would not otherwise have been captured by a single event. Location represents the largest predictor of the user region with 70.3%, but would lose 29.3% if used as a single feature. Users that posted a tweet referencing a local news media source captured 25.8% of users that was effective considering most people use Twitter for news purposes.^10^ This proved to be a unique and valuable asset to capture users propagating local news. Regional terms such as the “Ole Miss”, “USM”, and SMTTT played a slight increase in the data at 3.9%. There are no research articles that have currently utilized and validated the data in this approach. Previous papers have followed an approach solely on keywords or hashtags that have been less comprehensive.^11^ This comprehensive approach reveals the value of the triangulation to capture users.

**Exclusive Regional Criteria:**
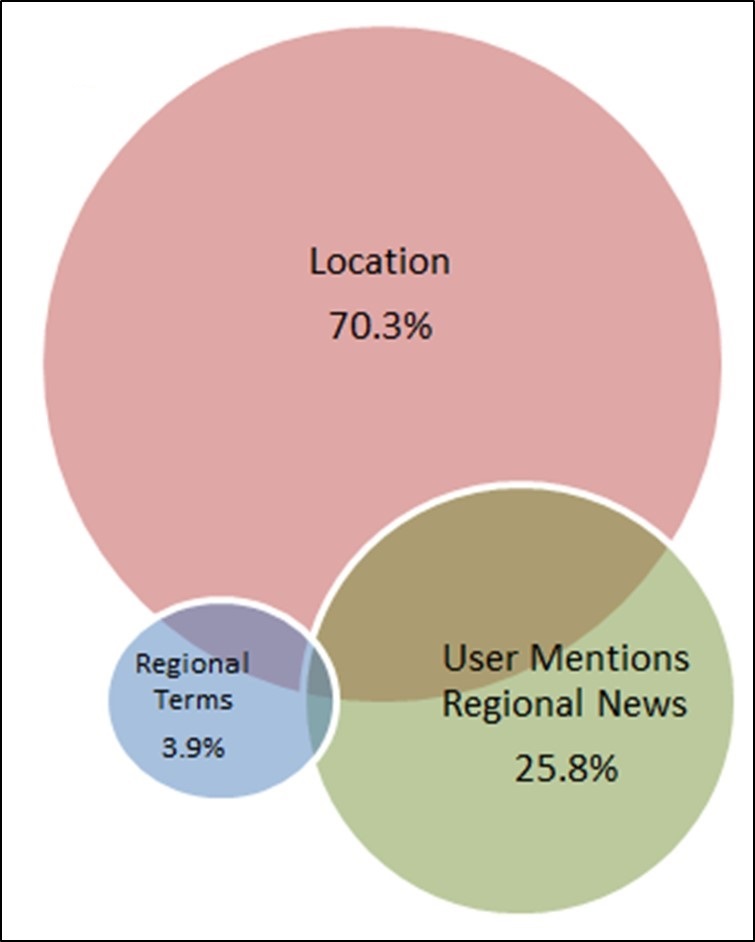
The percent of users that were captured by a single regional term.

Type of usage stayed consistent for both pre-storm and post-storm with mobile phones remaining over 70%; this shows the possible application of using the GPS feature during the time of a disaster and the mobility of this communication media in a disaster period. Prime hours of Twitter usage for the local population were displayed with the peak use times being during daytime hours and diminishing during the night time hours. (Figure 2-4) During the Tornado hours, a peak in Twitter usage was apparent with a 2-fold increase in tweets during this timeframe, yet mobile phone usage before and after the storm was consistent pre- and post-storm. This activity increased its outreach and ability to notify people without the need of sitting at a desktop computer (Note: merger of the top 6 devices used representing 60% of all tweet origination. The other 210 tweet programs are thinly distributed). Overall Twitter social media platform shows potential for disaster management and response to disseminate information to a local population group.

Currently, there are limited studies providing an easily accessible methodology to harnessing the power of social media. By being able to provide methodology to local or subset of a population group one can use social media locally and globally in many fields such as but not limited to: public health, information dissemination strategies, sociology, marketing, branding, political science, health information and behaviors, entrepreneurship, business, psychology, criminology, sex trafficking, drug trafficking, relationship cultivation strategies, linguistic studies, population studies, migration pattern studies, behavior studies, and educational technique; all of which were once inaccessible to the lay user.

Twitter’s unique features allow it to become a unique social media tool for emergency management and public health officials for rapid and accurate two-way communication.^12^ Additionally, understanding how and if a variable can be manipulated plays a crucial role in learning how to use this social media platform for effective, accurate, and rapid mass information communication. This knowledge will create a better framework for understanding how to create and alter messages that will be effectively received by the at-risk population in order to mitigate morbidity and mortality. This platform can be accessed via a smartphone so is relatively ubiquitous for all populations regardless of social and economic status.

The triangulation/ regional approach can be further adapted to real-time solutions based on the presence of an event within a region of the country. For example, bombing events could be regionally isolated based upon certain regional criteria, thereby zooming in on that part of the Twitter data stream to gain real-time analysis. Active and passive surveillance can be enhanced by coupling it with an artificial intelligence-like systems to monitor specific hashtags.^13^ This can potentially minimize the “noise” in twitter that would unlikely affect those outside the area. The triangulation approach could also be used to identify regional viral messages or superspreaders to get them on board with disseminating an impending threat at a grass roots level. This will be detailed further in Part III.

## Next Steps

Part III in our series describes the role of variables that may be purposefully manipulated (modified) to best use this social media platform for effective, accurate, and rapid mass information communication. This knowledge will create a better framework for understanding how to create and alter messages that will be effectively received by the at-risk population and mitigate overall morbidity and mortality outcomes. This platform can be accessed via a smartphone so is relatively ubiquitous for all populations regardless of social and economic status. The authors will also analyze the message itself and identify factors that promote risk communication and prevent morbidity and mortality at the local level during a time sensitive event.

## Limitations

Initial extraction must be performed based on criteria that bias the data to locally specific tweets, but independent access to the full Twitterverse database is prohibited by terms of service. To limit bias, the team utilized a comprehensive broad-based initial extraction. The findings are limited by the scope of time (96 hrs), and nature of the event that was monitored in the region. Regardless the pre-tornado activity was typical of most normal days and activities in the region. The team was was still able to extract and triangulate a total of 81,441 tweets and 10,646 Twitter users, 27,309 retweets, and 2,637 tweets with GPS coordinates (pre- and post-).

Search terms only utilized English and may miss minor misspellings of those terms not caught in the small user sampling. City names were not used outside of Birmingham and Hattiesburg due to corresponding cities in other states. Social media sites often provide anonymity that many users wish to preserve making defining their region impossible. Some users may be on business accounts. Some people view tweets without creating accounts.

User mentions are difficult to ascertain due to the name similarity between users. Pseudo-retweets or users who send a tweet designed to look like a retweet play a small role in the data, but present problems during filtration and extraction. Therefore, the actual population that received and responded to a tweet is likely an underestimate of the true population that received the message. Retweets are identical tweets that were messaged forward onto users. While one cannot truly measure whether an action was taken upon a Twitter communique an acknowledgment that a tweet was received and important enough to relay to its followers, retweets.

## Conclusions

This study describes the technically detailed methodological application of the novel triangulation methodology used to filter the haystack of tweets transmitted during the 2013 Hattiesburg Tornado among those captured from the over 2 billion tweets in the 96 hour window of the storm that were emitted on the Twitterverse. The data generated from the approach provides a descriptive analysis of the regional Twitter activity 48 hours pre- and post- Hattiesburg Tornado. By being able to target a subset of a population, rapid information dissemination is possible leading to a potential improvement in morbidity and mortality outcomes in local disasters.

## Competing Interests

The authors have declared that no competing interests exist.
